# Basic cytogenetics and physical mapping of 5S and 18S ribosomal genes in *Hoplias malabaricus* (Osteichthyes, Characiformes, Erythrinidae) from isolated natural lagoons: a conserved karyomorph along the Iguaçu river basin

**DOI:** 10.3897/CompCytogen.v8i3.7084

**Published:** 2014-08-12

**Authors:** Gisele Gemi, Roberto Laridondo Lui, Fernando Rodrigo Treco, Leonardo Marcel Paiz, Rafaela Maria Moresco, Vladimir Pavan Margarido

**Affiliations:** 1 Centro de Ciências Biológicas e da Saúde, Universidade Estadual do Oeste do Paraná, Rua Universitária 2069, CEP: 85819-110, Cascavel, PR, Brazil; 2 Universidade Paranaense, Avenida Julio Assis Cavalheiro 2000, CEP: 85601-000, Francisco Beltrão, PR, Brazil; 3 Universidade Estadual de Maringá, Departamento de Biologia, CEP: 87020-900 Maringá, PR, Brazil

**Keywords:** Chromosomal conservadorism, double-FISH, evolution, karyotype, rDNA

## Abstract

Erythrinidae include Neotropical teleost fish that are widely distributed in South America. *Hoplias* Gill, 1903 include two large groups: *H. malabaricus* Bloch, 1794 and *H. lacerdae* Miranda Ribeiro, 1908. *Hoplias malabaricus* is characterized by remarkable karyotype diversity, with some karyomorphs widely distributed geographically while others are more restricted to certain river basins. Cytogenetic analyzes were performed in a population of *Hoplias malabaricus* from the Wildlife Refuge of Campos de Palmas, the Iguaçu River basin. The specimens showed diploid number of 42 chromosomes (24m+18sm) without differentiated sex chromosomes system. The impregnation by silver nitrate showed multiple AgNORs. Seven pairs (4, 7, 10, 13, 16, 20 and 21) carrying 18S rDNA were detected by FISH. Heterochromatin was verified in the centromeric and pericentromeric region of most chromosomes and the terminal region of some pairs. FISH with 5S rDNA probes showed two chromosome pairs carrying these sites in the interstitial region (8 and 14). The data obtained in this study are similar to those found for two other populations of *H. malabaricus* already studied in the basin of the Iguaçu River, confirming the hypothesis that this species is natural, not having been introduced, as well as having an intrinsic characteristic, such as the largest number of sites of 18S rDNA.

## Introduction

The basin of the Iguaçu River, located in the southern region of the State of Paraná, is comprises a drainage area of 69,373 square km and a length of 1,275 km in its main riverbed. Its springs emerge from Serra do Mar and flow towards the First Plateau, or Plateau of Curitiba, and to the Second and Third Plateau. In the latter, the Iguaçu river basin is bordered by the Plateau of Palmas at the border of the State of Santa Catarina (Silva et al. 2001), where 79% belongs to the State of Paraná, 19% to the State of Santa Catarina and 2% to Argentina (Agostinho et al. 1997). The Iguaçu river basin has a low diversity of species and a high degree of endemism, with a total of 106 species, being 35 of Characiform, 46 of Siluriform and 11 Perciform (Baumgartner et al. 2012). This endemism has as its main cause the appearance of the Iguaçu Falls at the last part of the flow (Agostinho et al. 2004). In addition to this large geographical barrier of 72 meters, other barriers that segment the Iguaçu river were observed along its flow: Salto Caiacanga (9 meters), Salto Grande (13 meters), Salto Santiago (40 meters) and Salto Osorio (30 meters) (Maack 1981).

Erythrinidae are characterized by a sedentary lifestyle which consequently reduces gene flow between the populations that inhabit the same basin since they do not overcome obstacles, such as waterfalls (Blanco et al. 2010). This family is composed of three genera: *Erythrinus* Scopoli, 1777, *Hoplerythrinus* Gill, 1896 and *Hoplias* Gill, 1903 (Gayet et al. 2003). *Erythrinus* is comprised of two species, *Erythrinus erythrinus* Bloch & Schneider, 1801 and *Erythrinus kesslerie* Steindachner, 1877 (Oyakawa 2003). *Hoplerythrinus* includes three species: *Hoplerythrinus cinereus* Gill, 1858, *Hoplerythrinus gronovii* Valenciennes, 1847 and *Hoplerythrinus unitaeniatus* Spix & Agassiz (Froese and Pauly 2014). *Hoplias* is the most widespread in South America, composed of two large groups: *Hoplias lacerdae* Miranda Ribeiro, 1908 and *Hoplias malabaricus* Bloch, 1794, the first group containing six species (Oyakawa and Mattox 2009), and the second is a classic case of cryptic species related to chromosomal aspects (Bertollo et al. 2000).

According to Bertollo et al. (2000), *Hoplias malabaricus* is a Neotropical freshwater species widely distributed and with great karyotype diversity (different karyomorph). The chromosomal studies show diversity in diploid number from 39 to 42 chromosomes, differences in chromosomal formulas and presence (karyomorph B, D and G) or absence (karyomorph A, C, E and F) of a sex chromosome system. According to this author, *Hoplias malabaricus* includes seven karyomorph, being some of them more widely distributed, such as karyomorph A, C and F, and other restricted to only one or a few sites, such as karyomorph B, D, E and F. Populations of this species from the basin of the Iguaçu river were previously analyzed by cytogenetic methods and two of these karyomorph were detected (A and B) (Lemos et al. 2002, Vicari et al. 2003, Vicari et al. 2006). The karyomorph A appears to be more widely distributed throughout this basin, while the karyomorph B is restricted to only one population in a region next to its riverbed side (Lemos et al. 2002).

With regards to the occurrence of *Hoplias malabaricus* in the basin of the Iguaçu river, there is a controversy as to its origin in this location. According to Garavello et al. (1997), *Hoplias malabaricus* would not be a native species of the Iguaçu river, which may have been introduced from nearby basins. Subsequently, a study with chromosomal populations of this species suggested that the karyomorph A is a native form of the Iguaçu River. In addition, the little karyotype diversity detected among the populations that were analyzed must be due to vicariant events (Vicari et al. 2006).

In this sense, the objective of this work was to study - through cytogenetic techniques - a population of *Hoplias malabaricus* collected in a natural lagoon in the region of Palmas, in the far south of the State of Paraná – Brazil. This lagoon has no contact with other aquatic environments and is isolated from other river systems, in order to better understand the geographical distribution of the group in the basin of the Iguaçu river.

## Methods

**Figure 1.**
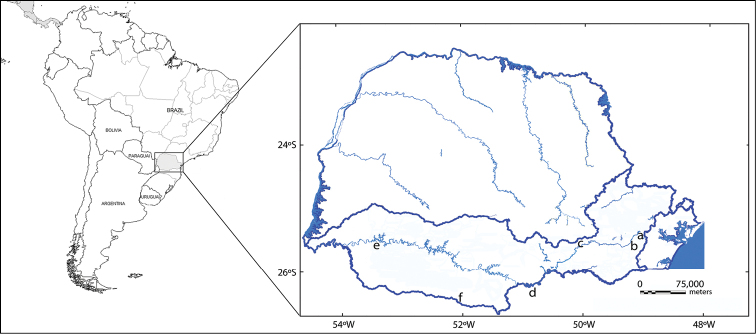
Map of sampling sites of *Hoplias malabaricus* populations in Iguaçu river basin: **a** Piraquara **b** São José dos Pinhais **c** Palmeira **d** Poço Preto **e** Nova Prata do Iguaçu and **f** Palmas (present paper).

Four specimens were collected (2 males and 2 females) of *Hoplias malabaricus* from isolated lagoons in the region of Palmas of the Wildlife Refuge of Campos de Palmas, in the Iguaçu river basin, belonging to the State of Paraná – Brazil (Fig. 1). This reduced sample is duet to the collections being made on a conservation unit, and a major sampling would be justified if intra- or interpopulational chromosomal polymorphisms were observed. The samples were anesthetized and sacrificed by an overdose of clove oil (Griffiths 2000) for the removal of the material for the cytogenetic study. The mitotic chromosomes were obtained from a cell suspension using the anterior portion of the kidney in accordance with the technique adapted by Bertollo et al. (1978) and Foresti et al. (1993). Thirty metaphases spreads from each fish were analyzed and ten of the best mitotic metaphases were used to measure karyotypes. For the AgNORs analysis, the impregnation by silver nitrate has been used based on the methodology of Howell and Black (1980), and to determine the distribution pattern of heterochromatin, C-banding with barium hydroxide was used, following the proposal of Sumner (1972) with modifications proposed by Lui et al. (2012). For the analysis of fluorescent *in situ* hybridization (FISH) 5S rDNA probes of *Leporinus elongatus* Valenciennes, 1850 (Martins et al. 2000) and 18S rDNA of *Prochilodus argenteus* Spix & Agassiz, 1829 were used (Hatanaka and Galetti 2004). Each one of them was marked, respectively, with digoxigenin-11-dUTP and biotin-16-dUTP (Roche). The detection and amplification of the hybridization signal was performed using antidigoxigenin-rhodamine for 5S rDNA (Roche) and avidin-FITC and anti-avidin-biotin for 18S rDNA (Sigma). FISH was performed according to Pinkel et al. (1986) and modifications suggested by Margarido and Moreira-Filho (2008). The best metaphases were captured in an Olympus BX60 photomicroscope with a digital camera DP71 and DPcontroller 3.2.1.276 software (Olympus). The FISH slides were analyzed with an epifluorescence photomicroscope under an appropriate filter. The chromosomes were arranged in groups classified in metacentric, submetacentric, subtelocentric and acrocentric, according to the calculation of arm ratio as proposed by Levan et al. (1964).

## Results

**Figure 2.**
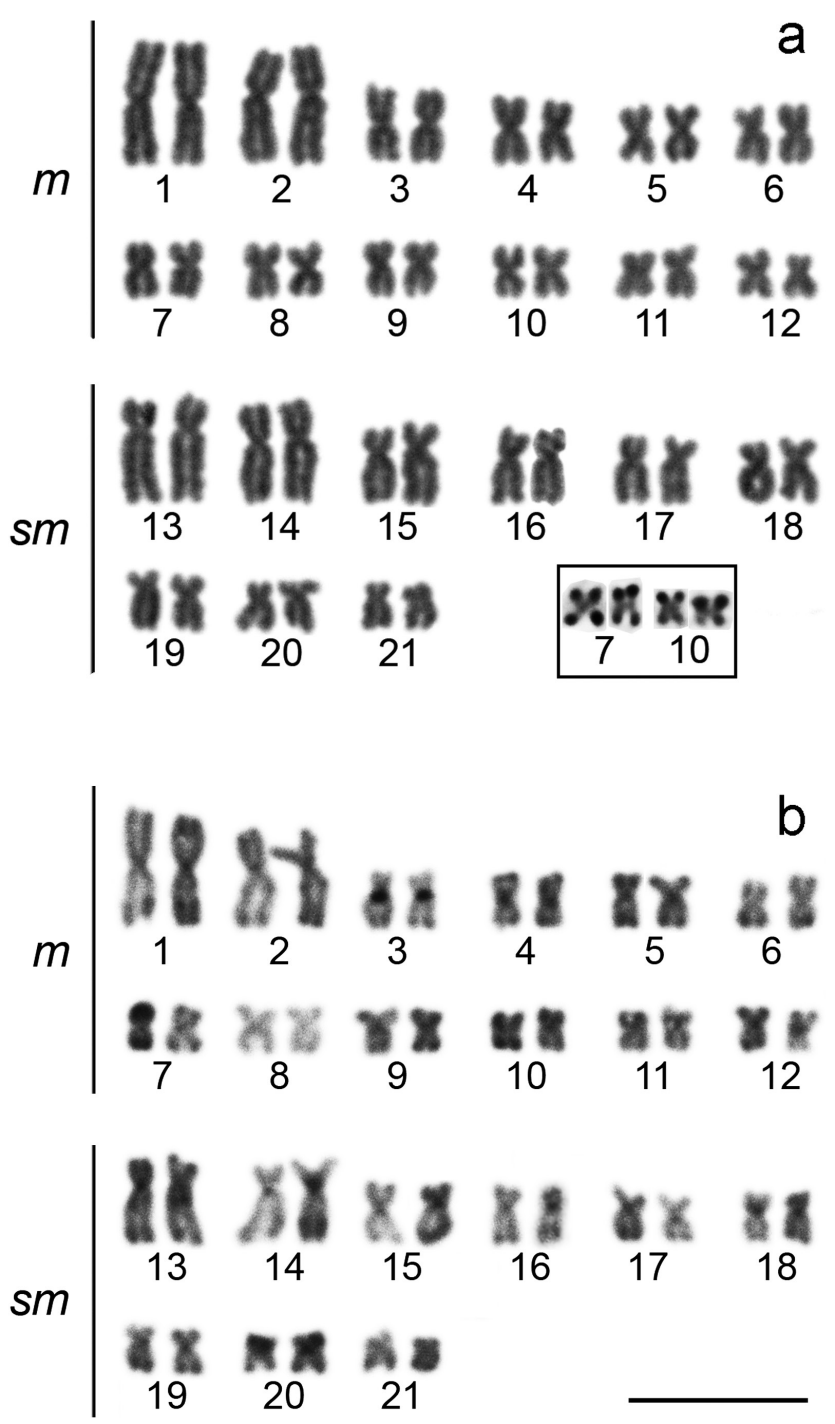
Karyotypes of *Hoplias malabaricus* stained with Giemsa (**a**) and treated through the C-banding (**b**). The AgNORs bearing chromosome pairs (7 and 10) are presented in box. Bar = 10 µm.

**Figure 3.**
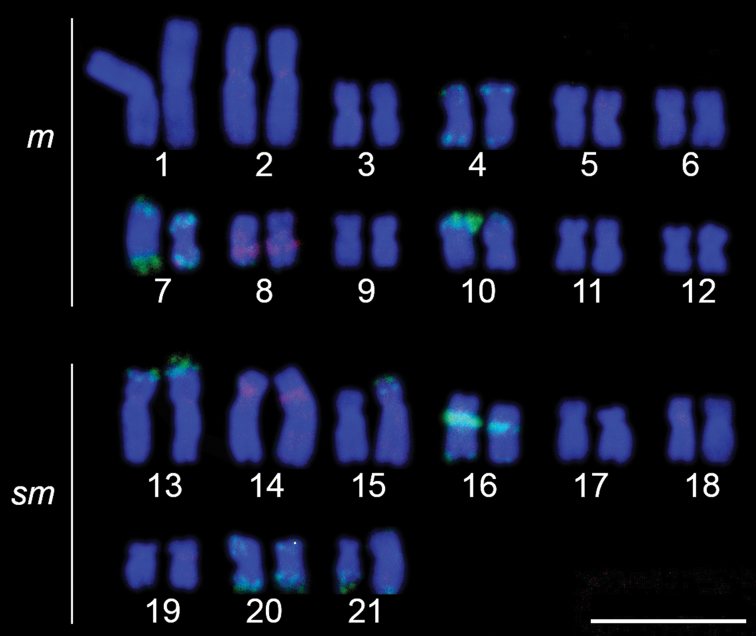
Karyotype of *Hoplias malabaricus* hybridized with 5S rDNA (digoxigenin, red) and 18S rDNA (FITC, green) probes. Bar = 10 µm.

The cytogenetic analysis observed diploid number of 42 chromosomes with 24 metacentric chromosomes and 18 submetacentric chromosomes, for male and female, and without a sex chromosome system (Fig. 2). The impregnation by silver nitrate showed multiple AgNORs, ranging from 4 to 6 NORs. The analyzed metaphases with silver nitrate impregnation presented bi-telomeric labels in the metacentric pair 7 and telomeric labels on the short arm of the metacentric pair 10 (Fig. 2, in box), coinciding with 18S rDNA, evidenced in FISH (Fig. 3). Five other pairs carrying rDNA 18S were marked by FISH, the metacentric 4 in both telomeric regions, the submetacentric pair 13 in the telomeric region of the short arm, pair 16 in the interstitial region of the long arm, pair 20 in both telomeric regions, and pair 21 in the terminal region of the long arm (Fig. 3). The C-banding revealed heterochromatin in the centromeric and pericentromeric region in most chromosomes of the complement, as well as bitelomeric and terminal heterochromatin in some chromosomes, these being coincident with the AgNORs (pairs 7 and 10) (Fig. 2). The FISH with 5S rDNA probe revealed two pairs of chromosomes, being interstitial on the long arm of the metacentric 8 and on the short arm close to the centromere of the submetacentric 14 (Fig. 3).

## Discussion

*Hoplias malabaricus* comprises a complex of species due to its wide karyotype diversity, and some karyomorphs are geographically widely distributed, while others have lower distribution and are restricted to certain basins, and even sympatric karyomorphs may occur without the detection of hybrids (Bertollo et al. 2000, Born and Bertollo 2006). The specimens analyzed showed chromosomal characteristics related to a diploid number, absence of a system of sexual chromosomes and karyotype formula that fits them in the karyomorph A of the *Hoplias malabaricus* group as designated by Bertollo et al. (2000). Previous studies in populations of *Hoplias malabaricus* from the Iguaçu river showed that this karyomorph is the most widely distributed in the basin (Fig. 1, Table 1). According to Blanco et al. (2010), who reviewed the chromosomal studies related to karyomorph A, a great part of South American river basins contain them, and multiple levels of chromosomal differentiation can be observed among allopatric populations (i.e., karyotype formula, heterochromatic distribution, AgNORs/18S rDNA, 5S rDNA and *5SHindIII* satellite DNA).

For the populations of karyomorph A of *Hoplias malabaricus* from the Iguaçu river, the karyotype formula does not show any clear marker to differentiate populations throughout this basin (Vicari et al. 2006) or to distinguish them from populations in neighboring basins (Ribera, Tibagi and Ivai) of the Iguaçu river (Vicari et al. 2005), which is different from what is observed for allopatric populations of several other regions (Blanco et al. 2010). Despite the paper of Lemos et al. (2002), bringing a slightly distinct formula (20m + 22sm), and the one from Bertollo et al. (2000) of not separating meta- and submetacentric chromosomes, our observation of these karyotypes suggests that they could be rearranged by 24 metacentrics and 18 submetacentrics, as detected by Vicari et al. (2003, 2005, 2006) and for the population of this study. It is worth noting that this conservation of the karyotype formula is not a common situation for populations distributed along this basin, as was already observed for *Astyanax altiparanae* Garutti & Britski, 2000, *Oligosarcus longirostris* Menezes & Géry, 1983, *Corydoras paleatus* Jenyns, 1842, *Pimelodus ortmanni* Haseman, 1911 and *Glanidium ribeiroi* Haseman, 1911 (Kantek et al. 2007). Furthermore, in relation to karyomorph A of *Hoplias malabaricus*, this level of conservation was not observed with other chromosomal markers.

The distribution of heterochromatin in all the karyomorphs of the *Hoplias malabaricus* complex has often been described in the terminal and pericentromeric region of some pairs of chromosomes (Dergam and Bertollo 1990, Haff et al. 1993, Bertollo et al. 1997a, 1997b, Born and Bertollo 2000, Vicari et al. 2005, Blanco et al. 2010), and was also observed in the population of the present study. However, a small variation in the amount and location of heterochromatin can be observed between the various allopatric populations already studied (i.e., Blanco et al. 2010). When the distribution of heterochromatin of the population in this study is compared to others of the Iguaçu river (Lemos et al. 2002, Vicari et al. 2006), or even with those that are present in the basins next to the Iguaçu (Vicari et al. 2005), a great similarity can be observed.

The analyses carried out by Vicari et al. (2006) in populations belonging to the basin of the Iguaçu river (Nova Prata do Iguaçu and Palmeira), demonstrated a variable number of AgNORs, usually located in the telomeric region, and bitelomeric AgNORs were also found in both populations, as for the population analyzed in this study. However, interstitial nucleolus organizing regions were observed on the long arm of chromosome pair 16 of the Palmeira population, this characteristic being uncommon for *Hoplias malabaricus*. Note that bitelomeric AgNORs have usually been found in *Hoplias malabaricus* (Bertollo 1996, Born and Bertollo 2001), being this characteristic considered a probable synapomorphy for the group (Vicari et al. 2006). Up to now, there is no evidence of populations belonging to karyomorph A of *Hoplias malabaricus* that do not have bitelomeric AgNORs (Blanco et al. 2010).

Only a population with hybridization data with 18S rDNA is described in literature regarding the Iguaçu river, with four pairs being detected, one pair with bitelomeric marking, one interstitial pair and two pairs with terminal marking. All these pairs of the previous study (Vicari et al. 2003, 2005) have chromosomes corresponding to the population of this study. In addition, other pairs showed sites carrying 18S rDNA (pair 4 and 20, bitelomeric; pair 13, terminal on short arm). More than one chromosome pair with 18S rDNA has already been detected for the karyomorph A of *Hoplias malabaricus* (Blanco et al. 2010). However, this is the first report of this last three pairs.

This study showed two pairs of 5S rDNA sites carrying chromosomes. Previous studies showed that this marker varies in number of sites among populations of karyomorph A, with a small metacentric pair with interstitial marking that seems to be conserved (Ferreira et al. 2007, Blanco et al. 2010), and a second pair (large submetacentric) that can be detected with interstitial marking on the short arm in a population of the Sao Francisco river (Blanco et al. 2010). The two pairs detected in the population of the Iguaçu River in this paper seem to be corresponding to these pairs mentioned above. Ferreira et al. (2007) compared the location of 5S rDNA sites in three karyomorphs of the *Hoplias malabaricus* group (A, D and F) and observed obvious differences between them, indicating that the number and distribution of sites are good markers of the Erythrinidae family, which shows the need for data for this marker in other populations of this basin, since the 5S rDNA data presented in this paper are the first related to the Iguaçu river basin.

With the uplift of the Iguaçu Falls, an effective geographic isolation was created for the ichthyofauna of the First and Second Plateaus in the largest part of the Iguaçu river (Maack 1981), resulting in a pronounced endemism of its ichthyofauna (Garavello et al. 1997). This endemism is proposed for several groups of fish, therefore the occurrence of *Hoplias malabaricus* in the basin could be due to human introduction (Sampaio 1988, Dergam et al. 1998). However, other explanations are considered as well relating to its presence in the basin. The karyomorph A of *Hoplias malabaricus* features a wide distribution throughout the southeast and south of Brazil, being present in several rivers in the State of Paraná, reaching Uruguay and Argentina (Bertollo et al. 2000). Due to the old shaping of the Iguaçu river basin and the broad distribution that has been detected for this karyomorph A, Vicari et al. (2006) proposed that this species may not have been introduced as was previously believed. The analysis of this population present in the city of Palmas reinforces this hypothesis, not only due to being another population of this karyomorph in the basin, but mainly because this population comes from a natural lagoon located in an isolated region that is part of the hydrographic system of the Iguaçu river.

Therefore, the population analyzed in this study showed the same diploid number, karyotype formula, lack of a differentiated sex chromosomes system when compared to other populations of the Iguaçu river, in addition to sharing some characteristics with respect to the number and location of AgNORs, distribution of heterochromatin and 18S rDNA sites. These data confirm the hypothesis that *Hoplias malabaricus* is natural to the Iguaçu River, and in spite of presenting some intrinsic characteristics of this population, it represents the same evolutionary unit along the basin, which is in the process of allopatric differentiation through the setting of small rearrangements in the microstructure.

**Table 1.** Cytogenetical data of *Hoplias malabaricus* populations from Iguaçu river basin.

Locality	Karyomorph	Karyotype formula	AgNORs	Heterochromatin (C-banding)	18S rDNA	5S rDNA	Reference
Piraquara municipality (PR)	A	20m+22sm	Multiple:- 2 to 6 chromosomes (1 bitelomeric pair)	Pericentromeric and interstitial	-	-	1
São José dos Pinhais municipality (PR)	B	24m+16sm+2st (XX/XY)	Multiple	Pericentromeric	-	-	1
Poço Preto municipality (SC)	A	42 m-sm	-	-	-	-	2
Palmas municipality (PR)	A	24m+18sm	Multiple:- pair 7, m, bitel- pair 10, m, tel, sa	Pericentromeric and terminal	- pair 4, m, bitel- pair 7, m, bitel- pair 10, m, tel, sa- pair 13, sm, tel, sa- pair 16, sm, int, la- pair 20, sm, bitel- pair 21, sm, tel, la	- pair 8, m, int, la- pair 14, sm, int, sa	3
Nova Prata do Iguaçu municipality (PR)	A	24m+18sm	Multiple:- 3 to 8 chromosomes	Pericentromeric and terminal	-	-	4
Palmeira municipality (PR)	A	24m+18sm	Multiple (2 to 7 chromosomes):- pair 10, m, bitel- pair 16, sm, int, la- pair 21, sm, tel, la	Pericentromeric and terminal	- pair 4, m, tel, la- pair 10, m, bitel- pair 16, sm, int, la- pair 21, sm, tel, la	-	4, 5, 6

PR: Paraná state, Brazil; SC: Santa Catarina state, Brazil; m: metacentric; sm: submetacentric; tel: telomeric; bitel: bitelomeric; int: interstitial; la: long arm; sa: short arm.References: 1 - Lemos et al. (2002); 2 - Bertollo et al. (2000); 3 - Present paper; 4 - Vicari et al. (2006); 5 - Vicari et al. (2003); 6 - Vicari et al. (2005)
